# P-704. Sustained decline in the incidence of hospital-acquired Influenza A/B, Parainfluenza, and Respiratory Syncytial Virus (RSV) infections at the NIH Clinical Center

**DOI:** 10.1093/ofid/ofae631.900

**Published:** 2025-01-29

**Authors:** LaToya A Forrester, Robin T Odom, Joseph Scaletta, Angela V Michelin, Alison Han, David K Henderson

**Affiliations:** National Institutes of Health Clinical Center, Bethesda, Maryland; National Institutes of Health, Bethesda, Maryland; National Institutes of Health Clinical Center, Bethesda, Maryland; NIH, Bethesda, MD; National Institutes of Health, Bethesda, Maryland; NIH, Bethesda, MD

## Abstract

**Background:**

Respiratory viral infections can result in significant morbidity and mortality for immunocompromised patients. The role of universal masking in hospitals to reduce the spread of respiratory infections remains controversial. The NIH Clinical Center serves immunocompromised patients and continued masking policies into 2024 (masking throughout the hospital April 8, 2020-June 4, 2023 and masking only in patient care from June 5, 2023 onward). We conducted a retrospective review of hospital-acquired Influenza A/B, Parainfluenza, and RSV infections from January 2010 to March 2024 to determine if masking policies were associated with a reduction in hospital transmission of selected respiratory viruses.

**Methods:**

Incubation periods for each respiratory virus was established. Infections occurring after the incubation periods were considered hospital-acquired. Medical chart reviews were conducted to ascertain date of symptom onset and history of respiratory viral infection. Quantitative data analysis was performed.

**Results:**

Between January 2010 and March 2024, 127 hospital-acquired Influenza A/B, Parainfluenza, and RSV infections were identified. Forty-five Influenza A/B, 42 Parainfluenza and 37 RSV infections were identified in the pre-masking period (January 2010 to April 7, 2020) vs. 0 Influenza A/B, 2 Parainfluenza and 1 RSV infections identified in the masking period (April 8, 2020 to March 31, 2024). Interrupted time-series analysis was significant for all hospital-acquired respiratory viral infections (p < 0.001).

**Conclusion:**

Masking was temporally associated with a decrease in all hospital-acquired respiratory viral infections. Pandemic mitigation measures, including symptomatic screening of building entrants, physical distancing, and asymptomatic testing of staff, may have contributed to this decline; however, when these strategies were relaxed and masking continued, hospital-acquired respiratory viral infections remained low. Wearing masks in the hospital may provide benefit for hospitalized immunocompromised patients.

**Disclosures:**

**All Authors**: No reported disclosures

